# Immunotherapeutic Approaches for Treating Hepatocellular Carcinoma

**DOI:** 10.3390/cancers14205013

**Published:** 2022-10-13

**Authors:** Wanying Shen, Yujie Chen, Pan Lei, Marisela Sheldon, Yutong Sun, Fan Yao, Li Ma

**Affiliations:** 1 Hubei Hongshan Laboratory, College of Biomedicine and Health, Huazhong Agricultural University, Wuhan 430070, China; 2 Hubei Clinical Research Center for Precise Diagnosis and Treatment of Liver Cancer, Taihe Hospital, Hubei University of Medicine, Shiyan 442000, China; 3 Department of Experimental Radiation Oncology, The University of Texas MD Anderson Cancer Center, Houston, TX 77030, USA; 4 Department of Molecular and Cellular Oncology, The University of Texas MD Anderson Cancer Center, Houston, TX 77030, USA; 5 The University of Texas MD Anderson Cancer Center UTHealth Houston Graduate School of Biomedical Sciences, Houston, TX 77030, USA

**Keywords:** hepatocellular carcinoma, immunotherapy, immune checkpoint inhibitors, CAR T cells, resistance, combination immunotherapy

## Abstract

**Simple Summary:**

The incidence of liver cancer is increasing worldwide. When detected early, the most common form of primary liver cancer (hepatocellular carcinoma, or HCC) can be treated with surgery and organ transplantation (when feasible). However, in most cases, HCC is detected at advanced stages, and the survival benefit of current treatments (e.g., systemic therapy with kinase inhibitors) is very limited. The advent of immune checkpoint inhibitors (ICIs) has changed the treatment paradigm for multiple types of cancer, including HCC. The success of ICIs, especially in combination with anti-angiogenic drugs, has extended survival times for a subset of patients with HCC and has stimulated further preclinical and clinical development of immunotherapies, not just ICIs, but also T cell therapy and oncolytic immunotherapy. Because the immunosuppressive tumor microenvironment in HCC often allows cancer cells to escape destruction by the immune system and develop resistance to immunotherapy, combinations with other agents that could sensitize HCC to immunotherapy are actively pursued.

**Abstract:**

Liver cancer is a life-threatening disease, and its incidence is increasing globally. The most common form of liver cancer is hepatocellular carcinoma (HCC). Approximately half of patients with HCC, especially those at advanced disease stages, receive systemic therapies, including the tyrosine kinase inhibitors sorafenib and lenvatinib. Over the past few years, immune checkpoint inhibitors (ICIs) have changed the landscape of HCC treatment. In particular, the combination therapy with atezolizumab (an anti-PD-L1 antibody) and bevacizumab (an anti-VEGF antibody) significantly improved survival benefits compared with sorafenib as a single agent, a finding that has stimulated further preclinical and clinical development of immunotherapeutic approaches for treating HCC. In addition to ICIs, oncolytic immunotherapy and adoptive T cell therapy have also emerged as immunotherapeutic strategies. A major challenge is that the tumor microenvironment of HCC is usually immunosuppressive, leading to immune escape and immunotherapy resistance. Hence, combination therapies that could sensitize HCC to immunotherapy have become a growing area of investigation. In this review, we summarize recent advances in HCC immuno-oncology and review immunotherapeutic strategies that are under development for treating HCC.

## 1. Introduction

The liver, which is the largest internal organ in humans, performs many crucial functions. Liver cancer, estimated to reach more than one million cases per year by 2025, is one of the most common cancers worldwide and is among the fastest-growing cancer types in Western countries [1]. According to the American Cancer Society (www.cancer.org; accessed in July 2022), the 5-year survival rate for patients with liver cancer in the United States is approximately 20%. When the disease is diagnosed at an early stage, the 5-year survival rate is higher (~35%); for patients with metastatic liver cancer, the 5-year survival rate is less than 5%. The subtypes of primary liver cancer include hepatocellular carcinoma (HCC), intrahepatic cholangiocarcinoma (iCCA), fibrolamellar carcinoma, and hepatoblastoma [2]. HCC accounts for ~90% of all liver cancer cases [1]. Chronic viral hepatitis (types B and C), alcohol consumption, and aflatoxin exposure are the main risk factors for liver cancer, whereas low-dose aspirin usage is associated with decreased risk of HCC [3,4].

Hepatic resection and liver transplantation are potentially curative for early-stage HCC [3]. Unfortunately, many patients with liver cancer are not eligible for hepatic resection, and the number of liver donors is quite small. For intermediate-stage HCC, transarterial chemoembolization (TACE) is a standard of care [1]. For advanced-stage HCC, the US Food and Drug Administration (FDA) approved the use of the tyrosine kinase inhibitor (TKI) sorafenib in 2007, and since then, systemic therapies have become available [5]. Over the past 5 years, three additional TKIs—lenvatinib, regorafenib, and cabozantinib—have received worldwide approval [6]. As first-line systemic treatment options for unresectable HCC, lenvatinib and sorafenib showed comparable survival benefits [7]. When compared with placebo, regorafenib and cabozantinib could extend overall survival as second-line treatment for HCC [8,9]. Over the past decade, the advent of immunotherapy has changed the way that cancers are treated. For patients with advanced-stage HCC, treatment with the immune checkpoint inhibitor atezolizumab (an anti-PD-L1 antibody) plus the angiogenesis inhibitor bevacizumab (an anti-VEGFA antibody) yielded better overall and progression-free survival outcomes than sorafenib as a single agent [10,11], representing a breakthrough in the management of HCC. In this review, we summarize the molecular mechanisms of HCC immune responses and discuss recent developments in HCC immunotherapy.

## 2. The Immune Microenvironment of HCC

**Figure 1:**
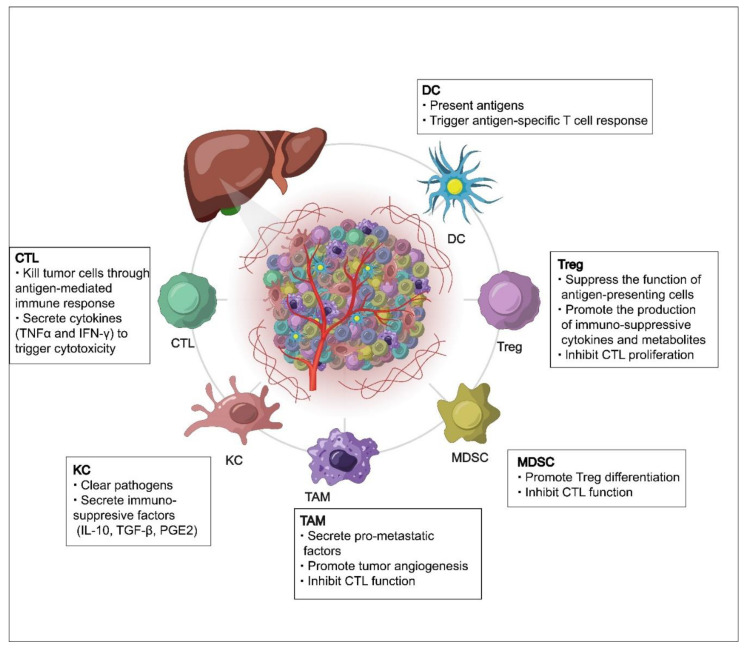
A schematic overview of the tumor immune microenvironment of hepatocellular carcinoma (HCC). Liver cancer cells and immune cells interact dynamically through cell–cell contact and the secretion and recognition of soluble factors, such as cytokines. Among various immune cells in the tumor microenvironment of HCC, regulatory T cells (Tregs), myeloid-derived suppressor cells (MDSCs), M2-type tumor-associated macrophages (TAMs), and Kupffer cells (KCs) suppress anti-tumor immunity, whereas cytotoxic T lymphocytes (CTLs) and dendritic cells (DCs) promote immune tumor rejection.

The tumor immune microenvironment plays pivotal roles in regulating tumor progression and therapy resistance [12]. In HCC, the immune microenvironment is dominated by immunosuppressive cells and signals that promote immune evasion and metastasis [13,14]. The main immune-suppressive cells in HCC are Kupffer cells, M2-type tumor-associated macrophages (TAMs), regulatory T cells (Tregs), and myeloid-derived suppressor cells (MDSCs) [13,14] (Figure 1). Kupffer cells are liver-resident macrophages that are responsible for the phagocytic clearance of pathogens [15,16]; on the other hand, Kupffer cells also induce T cell tolerance by secreting immunosuppressive factors such as interleukin (IL)-10, transforming growth factor (TGF)-β, and prostaglandin E2 [17,18]. During the progression of HCC, hepatic macrophages convert from an M1 phenotype to an M2 phenotype characteristic of cancer-promoting TAMs, which function as immune suppressor cells to support tumor growth, metastasis, and resistance to targeted therapy and immunotherapy [19,20]. Mechanistically, Kupffer cells and M2-polarized TAMs induce immune escape in HCC through the expression of PD-L1, the downregulation of MHC-II, the secretion of immunosuppressive cytokines, and the recruitment of Tregs and CD4^+^ cells [13,14,21]. Tregs, which are characterized by the expression of the transcription factor forkhead box p3 (Foxp3), promote immune tolerance in HCC by targeting effector T cells or by modulating antigen-presenting cells (APCs). It has been shown that Tregs can target APCs through the expression of negative regulatory cell surface receptors, such as cytotoxic T lymphocyte antigen 4 (CTLA-4), CD39, and CD73, or directly kill APCs by producing perforin and granzyme B [22]. In addition, Tregs can alter the immune microenvironment and suppress the immune response through the secretion of anti-inflammatory factors and the inhibition of IL-2 production by effector T cells [13,14,23]. MDSCs are usually present in cancer or other pathological conditions, but not in healthy individuals, although they are morphologically and phenotypically similar to neutrophils and monocytes. MDSCs can be classified into two groups: monocytic and polymorphonuclear. The monocytic MDSCs inhibit T cell responses through the production of anti-inflammatory cytokines and nitric oxide, whereas the polymorphonuclear MDSCs suppress T cells by generating peroxynitrite, which nitrates T cell receptors to reduce their responsiveness to antigen-MHC complexes [24,25,26].

In HCC, CD8^+^ cytotoxic T lymphocytes (CTLs), natural killer (NK) cells, and dendritic cells are the major cell types for immunosurveillance [27] (Figure 1). CTLs, also known as effector T (Teff) cells, kill cancer cells through granule exocytosis and Fas ligand-mediated apoptosis. CTLs also secrete interferon-γ (IFN-γ) and tumor necrosis factor α (TNFα) to trigger cytotoxicity in cancer cells. The activated T cells then start to express immune co-inhibitory receptors, such as programmed death-1 receptor (PD-1), which dampens the effector function of CTLs [28]. Similarly, NK cells exert their anti-cancer effects by secreting perforin and granulein to induce tumor cell apoptosis or by releasing pro-inflammatory cytokines and chemokines [29,30,31]. Dendritic cells are antigen-presenting cells that activate CTLs by presenting antigens to T cells and secreting immune co-stimulatory factors. Hence, dendritic cell vaccination against HCC may represent a promising immunotherapeutic strategy [27,32,33,34]. In addition, it has been suggested that M1-polarized macrophages may play a role in cancer immunosurveillance [35], but evidence supporting this role in HCC is still lacking.

## 3. HCC Immunotherapy

### 3.1. Immune Checkpoint Inhibitors

The immune checkpoint is an immune regulation mechanism by which immune co-inhibitory receptor signaling prevents strong immune responses from destroying healthy cells. Cancer cells exploit immune checkpoints to escape immunosurveillance. Several inhibitory immunoreceptors, including but not limited to PD-1, CTLA-4, T cell immunoglobulin, and mucin domain containing-3 (TIM3), lymphocyte-activation gene 3 (LAG3), and T cell immunoreceptor with immunoglobulin and ITIM domain (TIGIT), have been identified and characterized in cancer [36,37,38]. Immune checkpoint inhibitors (ICIs) target inhibitory immunoreceptor signaling to reprogram the tumor immune microenvironment from pro-cancer to anti-cancer, and many ICIs have been translated into therapies for cancer patients [36,37,38].

Several clinical trials have been carried out to test ICIs as single agents to treat HCC (selected mono-immunotherapy trials are listed in Table 1), some of which led to FDA approvals. Nivolumab and pembrolizumab were approved in 2017 and 2018, respectively, to treat patients with HCC who had been previously treated with sorafenib (i.e., for second-line treatment). Nivolumab is a fully human immunoglobulin G4 monoclonal antibody targeting PD-1. In a phase I/II trial (CheckMate 040), the objective response rate to nivolumab was 20% in patients with HCC [39]. However, a randomized multi-center phase III trial (CheckMate 459) indicated that first-line treatment with nivolumab did not significantly improve the overall survival of patients with advanced HCC when compared with sorafenib [40]. Pembrolizumab is a humanized antibody targeting PD-1. A phase II clinical trial (KEYNOTE-224) testing pembrolizumab as a second-line therapy demonstrated an objective response in 18 of 104 (17%) HCC patients who had previously been treated with sorafenib [41]. Subsequently, a phase III trial (KEYNOTE-240) showed that pembrolizumab improved progression-free survival and overall survival of Asian patients with advanced HCC who had previously been treated with sorafenib [42], supporting further evaluation of pembrolizumab as a second-line agent for HCC treatment. Moreover, camrelizumab (SHR-1210), another humanized anti-PD-1 antibody, showed manageable toxicity and an objective response rate of 14.7% (32 of 217) as a second-line agent in treating Chinese patients with advanced HCC, according to a multi-center randomized phase II trial [43]. In addition, tremelimumab is a humanized monoclonal antibody targeting CTLA-4 and is one of the earliest ICIs tested in clinical trials for treating HCC patients. Although a phase II trial demonstrated the safety of tremelimumab in HCC patients with chronic hepatitis C virus (HCV) [44], phase III trial data on tremelimumab for treating HCC patients with HCV are not yet available.

### 3.2. Chimeric Antigen Receptor (CAR) T Cells

CAR T-cell therapy involves engineering a cancer patient’s own T cells to recognize and eliminate tumor cells [48,49]. To date, the FDA has approved CAR T cells targeting CD19 or B-cell maturation antigen for treating blood cancers, including acute lymphoblastic leukemia, non-Hodgkin lymphoma, multiple myeloma, and relapsed or refractory large B cell lymphoma [50,51,52]. Although CAR T-cell therapy has not yet been approved by the FDA for the treatment of HCC and other solid cancers, CAR T cells targeting different tumor-associated antigens have been developed, and some have been tested in clinical trials. For example, glypican-3 (GPC3) is overexpressed in HCC, but shows little or no expression in normal tissues, making it an excellent HCC antigen for CAR T-cell therapy [53]. GPC3 CAR T cells were found to eliminate GPC3-positive HCC cells in mouse models [54], and a phase I clinical trial demonstrated its safety, as well as some early signs of anti-tumor activity of GPC3 CAR T cells in patients with advanced HCC [55]. CAR T cells have also been engineered to target other HCC-associated antigens, such as α-fetoprotein (AFP) [56], CD147 [57], CD133 [58], HBV surface protein [59], NKG2D [60], and c-MET [61]. These CAR T cells have yet to be tested in clinical studies. In addition, further identification of novel HCC-specific antigens may help to improve the safety and efficacy of CAR T-based immunotherapy.

### 3.3. Oncolytic Immunotherapies

Oncolytic immunotherapy involves the use of oncolytic viruses that are naturally occurring or genetically altered to kill tumor cells by selectively replicating in cancer cells, but not in normal cells [62]. Like CAR T-cell-based HCC therapy, oncolytic immunotherapy for treating HCC is still in the early development stages. In 2013, the Kim laboratory reported a phase I/II dose-finding clinical trial for oncolytic immunotherapy in liver cancer [46]. In this early trial, the vaccinia virus (Wyeth vaccine strain) known as JX-594, or Pexa-Vec, showed safety, oncolytic activity, and a dose-related survival benefit in advanced HCC. However, in a subsequent phase IIb trial, Pexa-Vec did not improve overall survival as a second-line therapy for patients with advanced HCC who had previously been treated with sorafenib [63].

### 3.4. HCC Vaccines

Therapeutic cancer vaccines are dendritic cell-based immunotherapies that achieve vaccination through the administration of tumor-specific antigens in combination with dendritic cell-activating adjuvants, or the administration of dendritic cells loaded with specific tumor antigens [64]. Hence, cancer vaccines activate the patient’s adaptive immune system to eliminate cancer cells. Among the various types of antigens, cancer researchers have turned their attention to tumor “neoantigens,” which arise from non-synonymous somatic mutations in the protein-coding regions, frameshift mutations, endogenous retroviruses, or tumor-specific post-translational modifications, such as phosphorylation and glycosylation [64]. Because of their tumor specificity and immunogenic characteristics, neoantigens have become attractive targets for developing cancer vaccines. Both neoantigen peptides and dendritic cells loaded with neoantigen have been developed to treat HCC. For instance, in a recent clinical study, treating HCC patients with personalized neoantigen peptides led to improved postoperative recurrence-free survival [65]. In a phase I trial that enrolled 17 HCC patients, intratumoral injections of the immune primer ilixadencel (pro-inflammatory allogeneic dendritic cells) were safe and elicited tumor-specific immunologic responses [47]. In a phase I/II trial that included 22 patients diagnosed with early- to immediate-stage HCC, intradermal administrations of HepaVac-101, a combination of peptide antigens and an RNA-based immunostimulator, showed safety and immunogenicity [66]. Further evaluations of clinical outcomes are warranted.

### 3.5. Immunotherapy Resistance in HCC

Despite promising clinical trials and recent FDA approvals of several immunotherapies, only a subset of HCC patients responded to this type of treatment [1,14]. Immunotherapy resistance in HCC can be attributed to the immune tolerance of the liver, as well as tumor-induced immune evasion [67]. As mentioned above, liver-resident macrophages, also known as Kupffer cells, are phagocytic cells that clear pathogens, and these cells also secrete immunosuppressive factors to induce tolerance to T cells [17,18]. In liver cancer, both tumor-intrinsic signaling, as well as crosstalk between cancer cells and the tumor microenvironment, contribute to immune suppression and immunotherapy resistance. For instance, HCC-intrinsic CDK20, also known as cell cycle-related kinase (CCRK), has been reported to induce immune escape through an NF-κB−EZH2−IL-6 axis [68]. HCC-secreted IL-6 recruits polymorphonuclear MDSCs, resulting in the suppression of T cell activity. In a preclinical model of liver cancer, inhibition of CCRK signaling led to increased sensitivity to anti-PD-L1 immunotherapy [68]. For HCC patients treated with ICIs, the activation of β-catenin signaling correlated with lower disease control rates, shorter progression-free survival, and shorter overall survival [69]. By using a transposon-mediated oncogene-induced HCC model, the Lujambio laboratory found that the activation of β-catenin signaling in HCC blocked CCL5 secretion, resulting in reduced dendritic cell recruitment, decreased T cell activity, and resistance to anti-PD-1 treatment [70]. In addition, the activation of PKCα−ZFP64−CSF1 signaling in HCC has been found to promote anti-PD-1 resistance via M2 macrophage polarization induced by CSF1 [71]. The rapid expansion of preclinical and clinical research on immune therapies for treating HCC is expected to reveal more insights into the mechanisms of immunotherapy resistance in HCC.

## 4. Combinatorial Therapeutic Strategies for HCC Treatment

**Figure 2:**
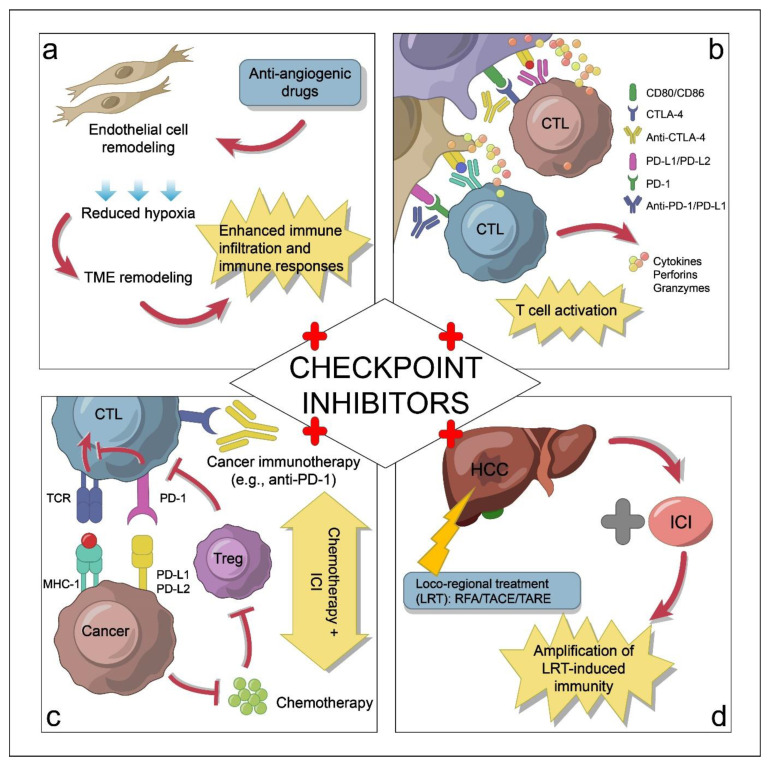
Schematic illustrations of how immune checkpoint inhibitors (ICIs) interact with other forms of treatment as combinatorial therapeutic strategies for hepatocellular carcinoma (HCC) treatment. (**a**). Combining ICIs with anti-angiogenic drugs (e.g., anti-VEGF) blocks HCC angiogenesis and boosts anti-tumor immune responses. (**b**). Dual ICIs that target non-redundant pathways of T cell inactivation (e.g., PD-1 and CTLA-4 pathways) outperform single-agent ICI therapy. (**c**). Adding chemotherapeutic drugs to ICIs promotes immunogenic cell death by activating dendritic cells, increasing T cell cross-priming, and suppressing myeloid-derived suppressor cells (MDSCs) and regulatory T cells (Tregs), which collectively boosts anti-tumor immunity. (**d**). Combining ICIs with neoadjuvant or adjuvant therapies can upregulate HCC antigens (e.g., glypican 3 and AFP), thereby enhancing immunotherapy efficacy. Abbreviations in this figure: CTL, cytotoxic T lymphocyte; MHC-1, major histocompatibility complex class I; RFA, radiofrequency ablation; TACE, transarterial chemoembolization; TARE, transarterial radioembolization; TCR, T cell receptor; TME, tumor microenvironment.

As mentioned above, single-agent immunotherapies have shown promising results in treating HCC, but response rates remain low. To overcome HCC immunotherapy resistance, it is beneficial to identify targets that can transform the HCC tumor microenvironment from immunologically “cold” to “hot,” thereby enhancing responsiveness to immunotherapy. In fact, a growing body of evidence has demonstrated the benefits of combinatorial therapeutic strategies for treating HCC (Figure 2 and Table 2).

Liver tumors usually contain multiple immunosuppressive factors, and blocking one factor is unlikely to substantially improve HCC treatment. Therefore, the simultaneous blockade of non-redundant pathways of immune suppression is expected to have better efficacy than the blockading of a single immune checkpoint. Indeed, in the CheckMate 040 trial (NCT01658878), the concomitant inhibition of the PD-1 and CTLA-4 pathways by nivolumab plus ipilimumab (an anti-CTLA-4 antibody) showed manageable safety and an objective response rate of 32% in patients with advanced HCC previously treated with sorafenib [72]. This combination therapy was approved by the FDA in 2020 [73]. In another study of HCC patients with disease progression on prior single-agent ICI treatment, dual ICI therapy with ipilimumab plus either nivolumab or pembrolizumab was found to achieve durable anti-tumor response and encouraging survival outcomes [74].

Because HCC is a highly vascularized cancer, targeting angiogenesis has become a promising therapeutic strategy. Currently, many clinical trials are testing angiogenesis inhibitors for HCC treatment, and a few of them have been approved by the FDA [76]. Vascular endothelial growth factor (VEGF) is not only a pro-angiogenic protein, but also an immune-suppressive factor [77]. It has been shown that VEGF suppresses immune responses by modulating cytotoxic T cells, dendritic cells, Tregs, and MDSCs [78,79,80]. Thus, one plausible approach is to combine immunotherapy with anti-angiogenic drugs to block HCC angiogenesis and boost anti-tumor immune responses. In patients with unresectable HCC, atezolizumab (anti-PD-L1) plus bevacizumab (anti-VEGF) was the first systemic therapy demonstrating an overall survival benefit surpassing that of sorafenib, based on a global open-label phase III trial, IMbrave150 (NCT03434379) [10,11,81]. This combination therapy was approved by the FDA to treat unresectable or metastatic HCC in 2020 [82]. A subsequent study identified potential biomarkers for clinical responses to this combination therapy, including high CD274, low GPC3 and AFP, high intratumor CD8^+^ T cell density, and low Treg/Teff ratio [83].

Therapies that combine ICIs with chemotherapy, radiotherapy, targeted therapy, or TACE are also under active clinical investigation. For instance, camrelizumab (anti-PD-1) combined with a standard FOLFOX4 (infusional fluorouracil, leucovorin, and oxaliplatin) regimen was used as a first-line treatment for advanced HCC in a multi-center phase Ib/II clinical trial, and this combination showed promising anti-tumor responses with good safety and tolerability [84]. That study led to a phase III trial (NCT03605706), now underway, comparing camrelizumab plus the FOLFOX4 regimen with the placebo plus the FOLFOX4 regimen for treating advanced HCC. In a phase Ib study (NCT03006926) that enrolled patients with unresectable HCC, the combination of lenvatinib (a multi-kinase inhibitor) with pembrolizumab showed promising anti-tumor activity with a manageable safety profile [75], and a phase III trial is now underway to compare this combination therapy with lenvatinib alone as a first-line treatment for advanced HCC (NCT03713593). In addition, several clinical trials are now ongoing to test combinations of immunotherapy with TACE for HCC treatment (e.g., NCT03778957, NCT04268888, and NCT04246177).

## 5. Conclusions

Immunotherapy has opened a new era in HCC treatment, and many clinical trials are underway to test ICIs and combination therapies in liver cancer. Compared with other systemic therapies, such as chemotherapy and targeted therapy, immunotherapy has unique advantages in managing advanced HCC. In particular, immune therapy has the potential to achieve systemic and durable anti-cancer responses via immunological memory, and this is clinically beneficial against HCC, which often exhibits multicentric and metachronous occurrences. Currently, the best available first-line therapy for advanced-stage HCC is the combination of the PD-L1 blockade with atezolizumab and the VEGF blockade with bevacizumab.

A major challenge for HCC immunotherapy is the unique immunosuppressive microenvironment of HCC, in addition to the immune tolerance of the liver itself. Despite recent breakthroughs in clinical trials and FDA approvals, only a small subset of patients with HCC respond to immunotherapy. Therefore, it is important to better understand the mechanisms of immune regulation in HCC and to identify biomarkers that predict immunotherapy response and guide the clinical use of ICIs and their combinations for HCC treatment. Compared with ICIs, CAR T-cell-based immunotherapies, oncolytic immunotherapies, and HCC vaccines are still in the early development stages. Future research should not only focus on improving the efficacy and safety of these immunotherapies, but also continue to identify HCC-specific antigens. Moreover, efforts should be made to identify new immune checkpoints and to develop novel immunotherapeutic agents, such as bispecific antibodies, antibody-drug conjugates, and agonist antibodies targeting immune co-stimulatory receptors. Ultimately, therapies that combine immunotherapies with other treatments are anticipated to improve clinical outcomes in advanced HCC.

## Figures and Tables

**Table 1:** Selected clinical trials of single-agent immunotherapy for HCC.

	Drug	Trial ID	Phase	*N*	Population	mOS	ORR	Reference
ICI	Nivolumab	NCT01658878	I/II	214	Advanced HCC	13.8	20%	[39]
		NCT02576509	III	371	Advanced HCC	16.4	15.4%	[40]
	Pembrolizumab	NCT02702414	II	104	Advanced HCC	12.9	17.3%	[41]
	Camrelizumab	NCT02989922	II	217	Advanced HCC	N/A	14.7%	[43]
	Tremelimumab	NCT02519348	I/II	69	Unresectable HCC	15.1	7.2%	[45]
Oncolytic virus	Pexa-Vec	NCT00554372	II	30	Unresectable HCC	6.7 (low dose), 14.1 (high dose),	15%	[46]
Vaccine	Ilixadencel	NCT01974661	I	11	Advanced HCC	2.7 (first-line), 10.9 (second-line)	N/A	[47]

6ORR, objective response rate; mOS, median overall survival (months); N/A, not available.

**Table 2:** Selected clinical trials of combinations of ICIs and other therapies for HCC.

Drug	Trial ID	Phase	*N*	Population	mOS	ORR	Reference
Nivolumab + ipilimumab	NCT01658878	I/II	49 (arm A)	Advanced HCC	22.8 (arm A)	32% (arm A)	[72]
Atezolizumab + bevacizumab	NCT03434379	III	336	Unresectable HCC	19.2	30%	[10,11]
Camrelizumab + FOLFOX4	NCT03605706	III	396	Advanced HCC	N/A	N/A	N/A
Pembrolizumab + lenvatinib	NCT03006926	Ib	104	Unresectable HCC	22.0	36%	[75]
Pembrolizumab + lenvatinib	NCT03713593	III	794	Advanced HCC	N/A	N/A	N/A
Nivolumab + TACE	NCT04268888	II/III	Recruiting	Intermediate stage HCC	N/A	N/A	N/A
Durvalumab + bevacizumab + TACE	NCT03778957	III	724	Locoregional HCC	N/A	N/A	N/A

ORR, objective response rate; mOS, median overall survival (months); N/A, not available.
